# Molecular Docking and In Silico Simulation of *Trichinella spiralis* Membrane-Associated Progesterone Receptor Component 2 (*Ts-MAPRC2*) and Its Interaction with Human PGRMC1

**DOI:** 10.1155/2022/7414198

**Published:** 2022-06-20

**Authors:** Muhammad Tahir Aleem, Asad Khan, Zhaohai Wen, Zhengqing Yu, Kun Li, Aftab Shaukat, Cheng Chen, Tauseef-ur -Rehman, Mingmin Lu, Lixin Xu, Xiaokai Song, Xiangrui Li, Ruofeng Yan

**Affiliations:** ^1^MOE Joint International Research Laboratory of Animal Health and Food Safety, College of Veterinary Medicine, Nanjing Agricultural University, Nanjing, Jiangsu, China 210095; ^2^Institute of Traditional Chinese Veterinary Medicine, College of Veterinary Medicine, Nanjing Agricultural University, Nanjing 210095, China; ^3^National Center for International Research on Animal Genetics, Breeding and Reproduction (NCIRAGBR), Huazhong Agricultural University, Wuhan 430070, China; ^4^Department of Parasitology, Faculty of Veterinary and Animal Sciences, The Islamia University of Bahawalpur, Pakistan

## Abstract

*Background*. *Trichinellosis* is a foodborne zoonotic disease caused by *Trichinella* spp., including *Trichinella spiralis*. This parasitic disease ranks as seven of the most infectious in the world. In this context, it is important to develop a vaccine that can combat *Trichinellosis*, especially for humans and pigs. This would be an important step in preventing transmission. In this study, we focus on homology modelling, binding site prediction, molecular modelling, and simulation techniques used to explore the association between *Trichinella spiralis* membrane-associated progesterone receptor component 2 (*Ts-MAPRC2*) and the human PGRMC1 protein. It was found that the progesterone receptor component 2 of *T. spirali*s has 44.54% sequence identity with human PGRMC1 (PDB ID: 4X8Y). Binding sites predicted for human PGRMC1 are GLU 7, PHE 8, PHE 10, PHE 18, LEU 27, ASP 36, and VAL 104. Molecular docking has six clusters based on *Z* scores. They range from -1.5 to 1.8. It was found that the progesterone receptor component 2 of *T. spiralis* has 44.54% sequence identity with human PGRMC1. During simulation, the average RMSD was 2.44 ± 0.20 Å, which indicated the overall stability of the protein. Based on docking studies and computational simulations, we hypothesized that the interaction of the proteins *Trichinella spiralis* membrane-associated progesterone receptor component 2 and human PGRMC1 formed stable complexes. The discovery of *Ts-MAPRC2* may pave the way for the development of drugs and vaccines to treat *Trichinellosis*.

## 1. Introduction

The zoonotic disease *Trichinellosis* is caused by a nematode parasite named *Trichinella spiralis* (*T. spiralis*). Among the most contagious parasitic diseases in the world, it ranks seventh [1]. The most common source of *T. spiralis* infection in humans is pork, commonly eaten raw, undercooked, or as a byproduct of pork processing [2–4]. Due to the prevalence of naturalized animal reserves in China, their high morbidity from this disease is becoming an increasingly serious problem due to the consumption of pork and pork products [5–7]. A major feature in the survival of *T. spiralis* nematodes is only through direct host-to-host transmission. It adapts to normal cellular functions and the immune system during all stages of infection [8, 9]. In spite of the wide use of antihelminthic agents against *Trichinellosis*, excessive use causes drug residues in meat, resistance to parasites, and other problems in the environment. In this light, the development of an effective vaccine to combat *Trichinellosis*, in particular for humans and pigs, would be an important step in preventing the transmission [7, 10]. As a vaccine applicant, a series of proteins have recently been discovered that inhibit host invasion and parasite viability and, thus, produce resistance against parasite infection. In addition, their ability to defend against *T. spiralis* larvae inoculation has been investigated in model animals [11–14]. Most of these vaccines have shown moderate success against *T. spiralis* infection; however, no comparable vaccine for *T. spiralis* infection is currently available [1]. Membrane-associated progesterone receptor (MAPR) proteins and progesterone receptor membrane component 1 (PGRMC1) and two (PGRMC2) are members of the same family. Porcine smooth muscle PGRMC1 protein of 28 kDa was the first to be collected [15–18]. Additionally, several studies have found that small androgen receptor-interacting proteins are found in cysts such as *S. japonicum* and that PGRMC receptors and progestin-induced proteins are found in helminths such as *S. japonicum* [19, 20]. The previous study focused on the cloning and characterization of the *T. spiralis* membrane-associated progesterone receptor component-2 gene (*Ts-MAPRC2*). A series of experiments throughout the process were performed, including expression, purification, immunoblot assay, binding ability against progesterone antibody, and immunofluorescence assay (IFA). Additionally, we assessed the direct effect of progesterone (P4) and mifepristone (RU486) on *Ts-MAPRC2* gene expression using in vitro cell culture tests that showed different levels of expression during all stages of development (muscle larvae, female adult worm, male adult worm, and newborn larvae). Afterward, mifepristone's in vivo phenotypic and relative mRNA effects on the F-AL stage were evaluated [21]. The current study focuses on the *T. spiralis* membrane-associated progesterone receptor component 2, and its interactions with human PGRMC1 have been docked and simulated in silico. Based on molecular docking and dynamic simulations, the study is now exploring the binding affinity of the membrane-associated progesterone receptor component 2 of *T. spiralis* and its interactions with human PGRMC1 to determine whether the compound has antiparasitic properties against *T. spiralis*.

## 2. Materials and Methods

### 2.1. Homology Modelling of *T. spiralis* Membrane-Associated Progesterone Receptor Component 2 (*Ts-MAPRC2*)

The amino acid sequence of *Trichinella spiralis* membrane-associated progesterone receptor component 2 (target protein) (NCBI accession no. XP_003375934.1) was submitted to the NCBI Blastp server https://blast.ncbi.nlm.nih.gov/Blast.cgi) to find the template protein in Protein Data Bank. The SWISS-MODEL server [22] with default parameters was used to model the three-dimensional structure of *Trichinella spiralis* membrane-associated progesterone receptor component 2. Two parameters were used to estimate the quality of the model: GMQE and QMEAN scoring. Additionally, the quality of the model was verified by the SAVES Server (https://saves.mbi.ucla.edu/). This server contains different algorithms to verify a model such as PROCHECK [23] which calculates the stereochemical quality of the model, ERRAT [24] which checks the interactions of noncovalently bonded amino acids, and VERIFY 3D which assesses compatibility of protein structure.

### 2.2. Retrieval of Human PGRMC1 Protein and Binding Site Prediction

The crystal structure of human PGRMC1 protein was retrieved from the Protein Data Bank (PDB ID: 4X8Y) and prepared in the PyMOL tool by removing the protoporphyrin and water molecules. After preparation of the PGRMC1 structure, the protein actives sites were predicted by using the CASTp server [25]. Similarly, the binding site residues of the model were predicted by the same method.

### 2.3. Molecular Docking

The molecular docking between the human PGRMC1 protein and *Trichinella spiralis* membrane-associated progesterone receptor component 2 was analyzed by the HADDOCK web server [26]. The resulting clusters were analyzed based on the *Z* score. The cluster with the best *Z* score was then selected to test the stability of the complex by using MD simulations. The protein-protein interactions were predicted by the KFC (knowledge-based FADE and contacts) web tool [26].

### 2.4. MD Simulation

The MD simulation of the selected cluster was conducted at 50 ns using the VMD [27] and NAMD [28]. The input files were prepared using AMBER21 tools [29]. The LeaP program was used to add missing hydrogen to protein [30]. The solvation of the protein-protein complex was done in a periodic box of 10 Å by using the TIP3P water model [31]. The system was neutralized by adding Na^+^ and Cl^−^ counter ions prior to the minimization step. The ff14SB force field was used for both proteins. To avoid energy clashes, the systems were relaxed by minimization at 10000 steps. After removing the clashes of systems, the solvation system was equilibrated at 310 K. Three additional equilibrations were run by increasing the temperature from 200 K to 250 K and 300 K to maintain the stability of the systems. Then systems were subjected to 50 ns simulation in a production run. The MD trajectories were stored every 2 ps during the production run. The trajectory analysis was carried out by VMD and BIO3D packages of R [32].

## 3. Results

### 3.1. Homology Modelling and Validation

**Figure 1:**
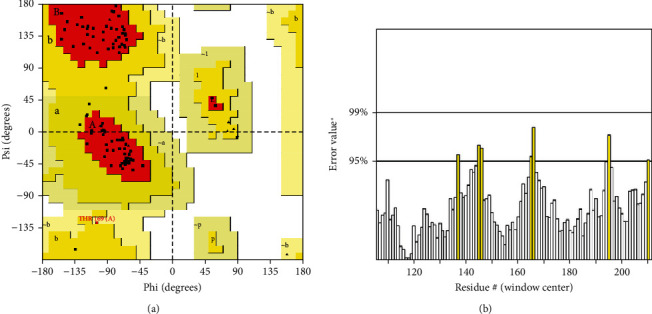
(a) Ramachandran plot. The residues in the red area are lying in the most favorable region (92.1%), while 6.9% of residues lie in additional allowed regions (yellow region). There was no residue in the disallowed region (white area). (b) ERRAT quality chart.

The membrane-associated progesterone receptor component 2 of *T. spiralis* showed a 44.54% sequence identity with the PGRMC1 of human (PDB ID: 4X8Y). The homology model had GMQE and QMEAN values of 0.36 and 0.64, respectively. To evaluate the modelling result, the GMQE combines properties from both the target-template alignment and the template search approach. Its value ranged from 0 to 1, with a greater number suggesting higher reliability of the predicted homology model. The QMEAN around zero suggested that the model structure and experimental structures of similar size were in good agreement, whilst scores of -4.0 or below indicated that the models were of poor quality. The PROCHECK software was used to assess the quality of the three-dimensional (3D) model by the Ramachandran plot (Figure 1(a)). It was observed that 93 amino residues (92.1%) were located in the most favorable region, 7 amino acids (6.9%) were located in the allowed region, and no amino acid was in a disallowed region which indicated the good quality of the model with 93.33 ERRAT quality factor (Figure 1(b)).

### 3.2. Binding Site Prediction

**Figure 2:**
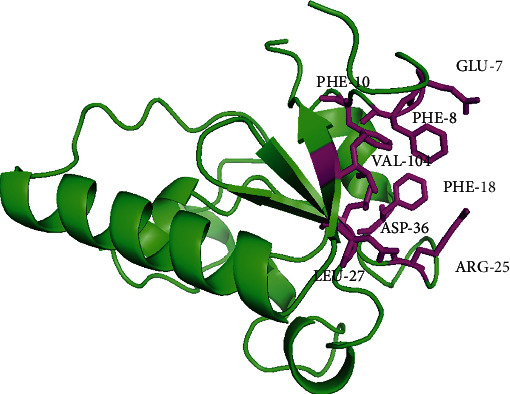
Predicted binding sites of human PGRMC1 shown in magenta sticks.

The binding sites of both proteins, i.e., human PGRMC1 and target protein, were predicted by the CASTp server. The predicted binding sites of human PGRMC1 were GLU 7, PHE 8, PHE 10, PHE 18, ARG 25, LEU 27, ASP 36, and VAL 104 (Figure 2) while all the residues of the target protein were predicted as binding site residues in during the prediction of the binding site. These predicted binding sites were used in the docking studies.

### 3.3. Molecular Docking

**Figure 3:**
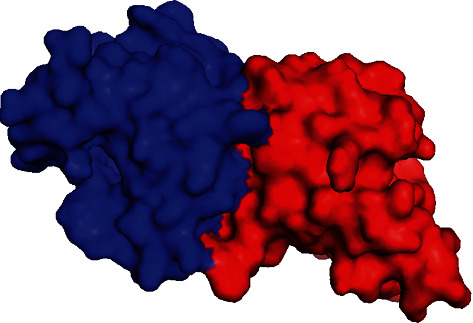
Target protein and human PGRMC1 protein cluster 4. The blue surface shows the human PGRMC1 protein while the red surface shows the target protein.

The molecular docking of a target protein and human PGRMC1 protein was performed by the HADDOCK 2.4 web server. The docking results are shown in (Table 1). There were a total of six clusters based on the *Z* scores. The *Z* scores range from -1.5 to 1.8. The cluster with a minimum *Z* score was selected for further studies. This cluster is shown in (Figure 3). The protein-protein interactions predicted by the KFC server are shown in (Table 2).

### 3.4. MD Simulation

**Figure 4:**
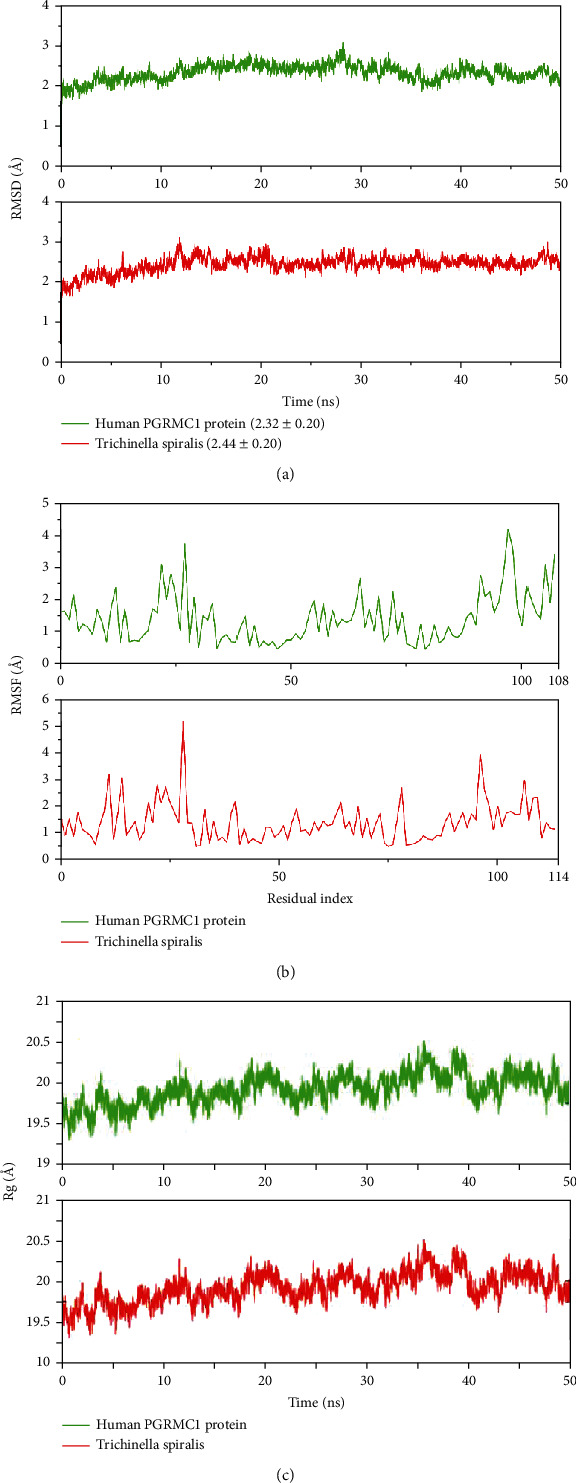
Protein-protein complex stability analysis by MD simulation. (a) RMSD plots. (b) RMSF plots. (c) Rg plots.

Cluster 4 was selected to check the binding stability in the complex form by a 50 ns simulation. For this purpose, different analyses, i.e., RMSD, RMSF, radius of gyration (Rg), dynamic cross-correlation, and principal component analysis (PCA), were performed.  Figure 4(a) shows the RMSD plots of the human PGRMC1 protein (green) and *Trichinella spiralis* (red). The RMSD plot of the human PGRMC1 protein showed that the RMSD of initial confirmation was ~2 Å at the start of the simulation. The RMSD remained in the range of ~2 to 2.5 Å till ~30 ns, and a slight increase was observed in value where it reached ~3 Å. The RMSD value attained stability after 30 ns and remained stable till the end of the simulation. The average RMSD value of this protein was 2.32 ± 0.20 Å which showed the overall stability of protein during simulation. Similarly, the RMSD plot of *T. spiralis* protein showed that the initial conformation of protein had an RMSD value of ~2 Å that increased to ~3 Å near ~12 ns. The RMSD value remained in the range of ~2.5 to 3 Å during 12 to 20 ns. After 20 ns, the RMSD value remained stable in the range of 2.5 to 3 Å till the end of the simulation. The average RMSD value of this protein was 2.44 ± 0.20 Å which showed protein stability. Similarly, the amino acid fluctuations were calculated by RMSF analysis.  Figure 4(b) shows the RMSF plots of the human PGRMC1 protein (green) and *T. spiralis* (red).

The green plot shows that the residues ~20-25 had the highest RMSF value which indicated that these residues showed maximum flexibility during the simulation. The remaining residues showed a lower RMSF value than these residues which showed that those were rigid during simulation except for the residues from ~90-108 as these are at the C-terminal. Similarly, the red plot showed the same behavior, and the residues from ~20-30 showed the flexibility which indicated the loop region in protein while the remaining residues with lower RMSF values showed the rigid part of the protein. The compactness of proteins was calculated by Rg. Rg calculates the protein unfolding events observed during the simulation.  Figure 4(c) shows the Rg plots of the human PGRMC1 protein (green) and *T. spiralis* (red). The Rg plots of both proteins showed the same trend throughout the simulation. The Rg value at the start was ~19.5 Å, increased to ~20.2 Å at 10 ns, and then dropped to ~20 Å after 12 ns. From 10 to 35 ns, the Rg value remained in the range of ~19.75 to 20 Å. After 35 ns, the Rg value increased to ~20.5 Å and remained in this range till 40 ns. The Rg value dropped to ~20 Å at 40 ns and then remained in this range till the end of the simulation. The Rg plots of both proteins showed that there were no unfolding events were observed during the simulation.

**Figure 5:**
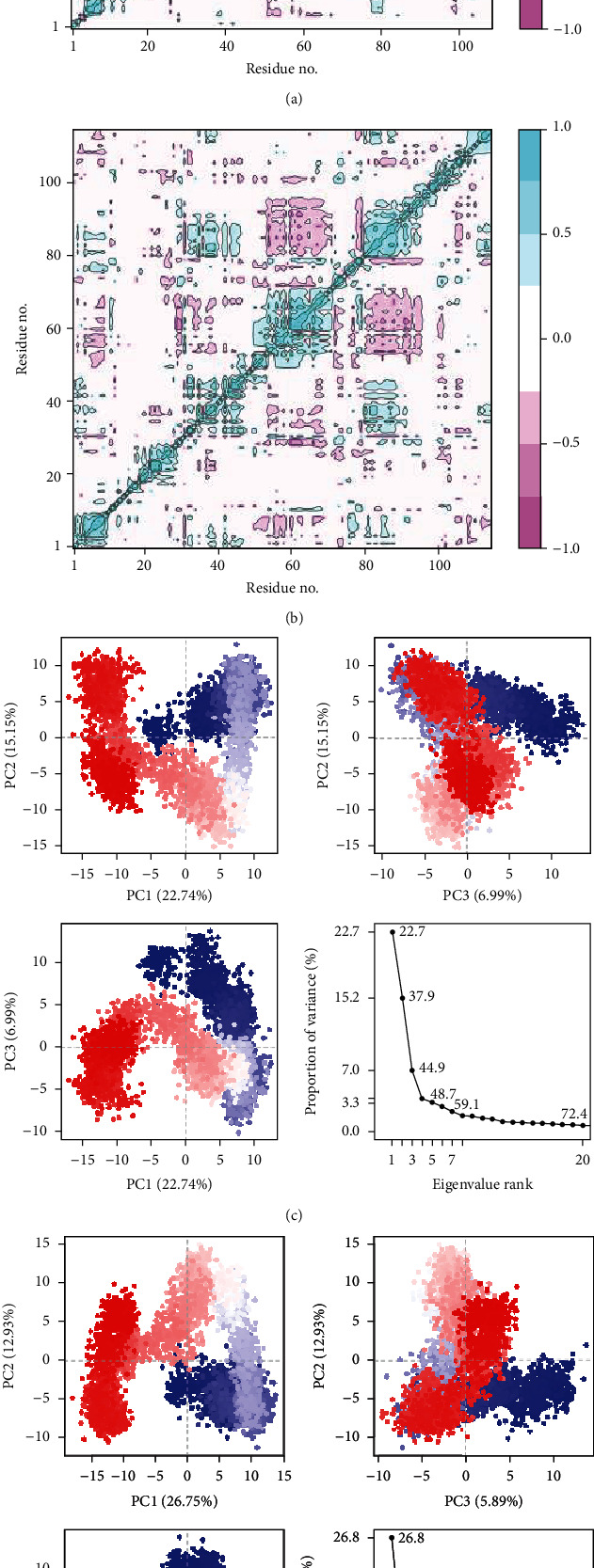
The dynamic motions analysis by simulation. (a) Cross-correlation map of human PGRMC1. (b) Cross-correlation map of the target protein. (c) PCA plot of Human PGRMC1. (d) PCA plot of the target protein.

The pairwise correlated motions among the residues of human PGRMC1 protein and *T. spiralis* were obtained from the trajectory. The cross-correlation maps of both proteins are shown in Figures 5(a) and 5(b), respectively. The cyan color showed the motions of correlated residues while the magenta color represented the anticorrelated residues. The solid diagonal cyan line showed the positively correlated residues of the protein during simulation. The dynamic motions of the residues were further investigated by the principal component analysis. In the graphs of both proteins, the eigenvalues of the protein had been plotted against the corresponding eigenvector index for the first twenty modes of motion (Figures 5(c) and 5(d)). The eigenvalues showed eigenvector fluctuations in hyperspace.

The overall movement of the proteins was controlled by eigenvectors with higher eigenvalues. The largest motion of the residues of human PGRMC1 protein was observed in PC1 (22.74%). The second most important direction was PC2 which accounted for a total of 15.15% variance in the protein residues. PC2 was orthogonal to PC1. Similarly, PC3 recorded a 6.99% variance. The total protein variance in the first three PC components was 44.88%. The overall variance in the *T. spiralis* protein was 45.57%. The highest motions recorded in PC1 were 26.75%, while the variations in PC2 and PC3 were 12.93% and 5.89%, respectively.

## 4. Discussion

PGRMC1 and PGRMC2 are membrane-associated progesterone receptor (MAPR) proteins that belong to the same family [16, 17]. In this study, we find the association of *Trichinella spiralis* membrane-associated progesterone receptor component 2 with human PGRMC1 protein by applying molecular modelling and simulation techniques. For this purpose, the homology model of the *Trichinella spiralis* membrane-associated progesterone receptor component 2 was built by SWISS-MODEL, and the accuracy of the model was validated by the Ramachandran plot and ERRAT quality score [33, 34]. The Ramachandran plot (Figure 1(a)) illustrated that the amino acid residues of the model were in the allowed region and not a single residue was in the disallowed or unfavorable region which explains the better quality of the model. To find the interactions of this protein with human PGRMC1 protein, the binding sites of both proteins were predicted by CASTp web server [35]. As a result, we find that the binding site residues of human PGRMC1 protein were GLU 7, PHE 8, PHE 10, PHE 18, ARG 25, LEU 27, ASP 36, and VAL 104 (Figure 2), while the target protein showed the whole sequence as the binding pocket. Both proteins were docked through the HADDOCK web server [36]. We prioritized the docked clusters based on the HADDOCK analysis and *Z* scores. We also discovered that the interactions between *Trichinella spiralis* membrane-associated progesterone receptor component 2 and human PGRMC1 protein had a KFC > 1, indicating that the best protein-protein interactions actually occurred in the conserved region [37]. The docking results showed that the closest distances between these two proteins were located in cluster 4, indicating that interactions are possible.

We further analyzed the details of proteins and their stability in the complex form through molecular dynamics simulation. Several analyses have been performed to check the stability of both proteins involved; root means square deviations, root means square fluctuations, the radius of gyration studies, dynamic cross-correlation maps, and principal component analysis. The RMSD analysis confirmed the steady confirmation with an average RMSD of 2.32 ± 0.20 and 2.44 ± 0.20 for human PGRMC1 and *Trichinella spiralis* membrane-associated progesterone receptor component 2 proteins, respectively. The residual flexibilities of both proteins were lower which showed that the hydrogen bonds of the systems maintained the integrity of protein structures during the simulation. Additionally, the steady radius of gyration plots of both proteins illustrated that the protein structures remained compact during the simulation. To further validate the complex stability, two additional analyses have been performed that explained the dynamic behavior of proteins throughout the simulation period. The cross-correlation maps of both proteins (Figures 5(a) and 5(b)) described the dynamic motions of proteins; a solid cyan diagonal line explained the positive correlation of residues while the magenta patches showed the negative correlations. The amino acid residues of both proteins were positively correlated throughout the simulation. Similarly, we performed a principal component analysis to calculate the variance in the residues in three hyperspace regions. The overall variance of 44.88% and 45.57% for human PGRMC1 and *Trichinella spiralis* membrane-associated progesterone receptor component 2 proteins, respectively, explained that the proteins were less flexible during simulation [21]. Hence, we assumed that both proteins made stable complexes based on the results of a simulation.

## 5. Conclusion

To conclude based on docking and simulation results, we assumed that the interaction of both proteins *Trichinella Spiralis* membrane-associated progesterone receptor component 2 and human PGRMC1 formed stable complexes. The discovery of this protein (*Ts-MAPRC2*) may open up new avenues of drug and vaccine development for the treatment of *Trichinellosis* in humans. Further studies are ongoing to determine the antibody effect of this protein (*Ts-MAPRC2*) in vitro and in vivo.

## Figures and Tables

**Table 1:** HADDOCK clusters of target protein and human PGRMC1 protein.

Clusters	Haddock score	Size	RMSD	Van der Waals energy	Electrostatic energy	Desolvation energy	Restraint's violation energy	Buried surface area	*Z* score
4	−49.8 ± 12.3	11	1.1 ± 0.9	−66.6 ± 3.0	−492.9 ± 57.0	−4.0 ± 1.6	1194.0 ± 81.8	2592.6 ± 165.3	-2.2
2	−19.1 ± 8.6	16	9.3 ± 0.1	−60.6 ± 6.6	−386.4 ± 20.9	2.5 ± 4.6	1162.5 ± 47.2	2317.2 ± 132.8	-0.7
9	−16.4 ± 18.1	6	13.4 ± 0.1	−65.6 ± 5.5	−224.1 ± 16.8	−21.5 ± 3.7	1155.6 ± 122.8	2005.8 ± 95.4	-0.6
15	−14.1 ± 19.3	4	11.8 ± 0.2	−54.5 ± 10.1	−348.1 ± 18.6	−14.0 ± 1.7	1240.1 ± 179.0	2317.9 ± 285.9	-0.5
1	−5.6 ± 10.0	26	14.9 ± 0.0	−62.2 ± 1.8	−180.4 ± 31.1	−18.3 ± 1.2	1109.5 ± 118.6	1866.2 ± 19.6	-0.1
8	0.6 ± 9.4	7	11.5 ± 0.1	−56.2 ± 5.1	−209.2 ± 34.9	−14.0 ± 2.7	1125.9 ± 101.5	1919.1 ± 41.5	0.2

**Table 2:** Protein-protein interactions based on KFC.

Chain	Residue	Number	KFC configuration value
A	PHE	8	HS (0.90 and 0.29)
A	PRO	24	HS (0.47)
A	ARG	25	HS (0.27)
A	ILE	26	HS (0.84 and 0.29)
A	ASP	36	HS (0.52)
A	THR	38	HS (1.18)
A	LYS	39	HS (1.20 and 0.28)
A	ARG	41	HS (1.38 and 0.28)
A	LYS	42	HS (0.24)
A	THR	98	HS (0.14)
A	HIS	102	HS (1.68 and 0.19)
A	VAL	104	HS (0.31)
B	ASN	125	HS (0.65)
B	ASP	127	HS (0.54)
B	LEU	173	HS (1.21 and 0.24)
B	ALA	174	HS (0.05)
B	LEU	176	HS (0.49 and 0.14)
B	ILE	181	HS (1.17 and 0.17)
B	LEU	184	HS (0.96 and 0.25)
B	ARG	185	HS (1.60 and 0.35)
B	MET	189	HS (1.23 and 0.21)
B	LYS	205	HS (1.64 and 0.37)
B	LEU	206	HS (0.47 and 0.23)
B	ASP	211	HS (0.19)

## Data Availability

Data is contained within the article.
